# Supplementary material for: A Novel Hydrophilic, Antibacterial Chitosan-Based Coating Prepared by Ultrasonic Atomization Assisted LbL Assembly Technique
Source: J Funct Biomater. 2023 Jan 12;14(1):43. doi: 10.3390/jfb14010043 (PMC9863527; doi:10.3390/jfb14010043)
Supplement: Supplementary file 1 [file jfb-14-00043-s001.zip › jfb-2078293-supplementary.pdf]

A Novel Hydrophilic, Antibacterial Chitosan-Based Coating Prepared by Ultrasonic Atomization Assisted LbL Assembly Technique

Xiaoyu Wang [1,†], Yuyang Zhou [2,3,†], Melissa Johnson [1], Cameron Milne [1], Sigen A [1], Yening Li [2], Wenxin Wang [1], Nan Zhang [2,*] and Qian Xu [1,*]

1 Charles Institute of Dermatology, School of Medicine, University College Dublin, D04 V1W8 Dublin, Ireland
2 Centre of Micro/Nano Manufacturing Technology (MNMT-Dublin), School of Mechanical and Materials Engineering, University College Dublin, D04 KW52 Dublin, Ireland
3 National Engineering Laboratory for Modern Silk, College of Textile and Clothing Engineering, Soochow University, Suzhou 215123, China
* Correspondence: nan.zhang@ucd.ie (N.Z.); qian.xu@ucd.ie (Q.X.)
† These authors contributed equally to this work.

Contents

Supplementary table S1, S2

References

Table S1. Element analysis results of CTS-CHO.

Sample	C (%)	N (%)	H (%)	Oxidation Degree (OD, %)
Chitosan (CTS)	41.64	7.22	6.49	N/A
Aldehyde chitosan (CTS-CHO)	37.81	5.27	5.5	12.53

Table S2. Zeta potential results of CTS solution and CTS-CHO solution.

**Sample**	**Zeta Potential (mv)**
1 mg/mL chitosan (CTS) solution	10.9
10 mg/mL aldehyde chitosan (CTS-CHO) solution	28.8
